# Successful Removal of Both Ovaries

**Published:** 1866-08

**Authors:** A. Reeves Jackson

**Affiliations:** Stroudburg, Pa.


*Successful Removal of both Ovaries.* By A. REEVES JACKSON, M. D., Stroudburg, Pa.

Sarah T., aged forty-seven years, unmarried, applied for relief, November 25, 1862. She commenced menstruating at the age of seventeen, and the function has always regularly and painlessly performed. Six or seven years ago, after having taken a severe cold, she felt a sensation which she compared to the occasional slow dropping of water in the region of the bladder. Shortly after this she discovered that the lower portion of her abdomen was enlarged, and that the increase was greatest on the left side. She also felt, indistinctly, a tumor about the size of a small orange in the same locality. In a single night the whole of this enlargement, including the tumor, spontaneously disappeared. However, it soon returned, and she has since been increasing gradually in size. Her complexion is dull and sallow, appetite good, bowels regular, although latterly she has experienced some difficulty in defecation, and also in urinating. The abdomen measures forty-one inches in circumference; is very greatly and equably distended, and distinctly fluctuating, the fluid evidently encysted. No solid tumor is discoverable through the abdominal walls. The left leg and foot are œdematous.

A viginal examination reveals a hard, smooth, elastic tumor, immovable or nearly so, completely filling the cavity of the pelvis. The os uteri circular, and with soft lips, is distinguished with difficulty. An attempt to introduce the sound is unsuccessful, the instrument appearing to meet with some obstruction immediately after entering the os.

Various remedial means were used without relief, and the oppression of the breathing, the swelling of the lower extremities, and other symptoms dependent upon the distension of the abdomen having become insupportable, tapping was decided upon, and performed January 1, 1863, with great and immediate relief to the patient. The fluid removed was glairy, of a pale straw color, somewhat tenacious, and measured forty-three pints. After its withdrawal the outlines of a solid tumor, occupying the lower portion of the abdomen and extending as high up as the umbilicus, were clearly distinguished, the greatest bulk being apparently on the left side. It was slightly movable, smooth, imperfectly globular, and without evident fluctuation.

The patient remained comfortable several weeks, but the abdomen again became distended, and tapping was repeated December 24, 1863. She was subsequently tapped several times, the intervals between the operations became each time shorter, and the last operation being done July 27, 1865.

The patient's general health now began rapidly to fail, and after having the dangers of the operation fully explained to her, she concluded to submit to ovariotomy.

The operation was performed October 31, 1865, in the presence and with the assistance of Drs. Bond, Bush, Barnes, Welling, Davis, Williams, Walton, and Mr. Van Buskirk, medical student. The patient having been well purged in the morning by an ounce of castor oil, was placed upon a narrow table, the nates brought near to the lower end, and her feet resting upon a chair without a back. Everything being in readiness, she was put under the influence of an anæsthetic mixture composed of one part of chloroform and two parts of sulphuric ether by measure. An incision four inches in length was made in the linea alba, commencing one inch below the umbilicus, and extending directly downward. This was gradually and cautiously deepened with the view of exposing and tapping the cyst and removing its fluid contents through the canula. The design was frustrated, however, by the knife suddenly entering the cyst and giving exit to sixteen pints of a gelatinous, whitish fluid. In order to facilitate the evacuations of the latter and to guard against its entrance into the peritoneal cavity, the patient was turned completely on her right side. The cyst having been emptied, it was ascertained that its walls were adherent to the peritoneum at the place of incision, and it was with very great difficulty that the adhesions could be sufficiently broken down to enable the hand to be introduced in order to learn the further condition of the parts involved. When this was finally accomplished it was found that there were two tumors entirely distinct, the one arising from the right, and the other from the left side. The one on the right side was a unilocular cyst with thickened walls, firmly adherent to the mesentery, omentum, intestines, and to the abdominal surface of the peritoneum. It was now empty and collapsed, and was maintained in its position by means of these numerous attachments.

The other, arising from the left side, was hard, firmly adherent to the peritoneal surfaces of the left abdominal and pelvic walls, and to the posterior and left sides of the fundus of the womb. The incision in the abdominal walls having been made sufficiently large, the attachments of the tumor were carefully separated by means of the fingers aided occasionally with the handle of the scalpel. The tumor was then lifted through the opening, and a clamp having been placed firmly upon the pedicle, the tumor was separated and removed. The pedicle, which consisted of the broad ligament of the uterus, was not more than one inch in length, about the thickness of a finger, and somewhat flattened. The empty cyst of the other tumor was next cautiously separated from its many attachments and drawn away. Its pedicle, which also consisted of the broad ligament, was about four inches wide, two and a half inches long, and traversed by large vessels. A clamp was placed upon it, and it was then cut close to its attachment to the tumor. The cavity of the abdomen was next carefully cleansed by means of newly prepared sponges and warm water, and the upper portion of the wound drawn together and retained by means of hare-lip pins and sutures, care being taken to bring the peritoneal surfaces accurately in contact. The clamps being found to interfere very greatly with the approximation of the edges of the lower portion of the wound, owing to the shortness of the pedicles, it was decided to remove them, and this was accordingly done, after previously securing the pedicles by strong ligatures applied beneath the clamps.

The remainder of the wound was then closed by iron-wire sutures and hare-lip pins. The pedicles, being drawn out by their ligatures, were transfixed by large hare-lip pins passed also through the edges of the wound, and thus kept on the surface of the abdomen. The stumps beyond the ligatures were freely touched with a solution of persulphate of iron. Strips of adhesive plaster were then placed across the abdomen, and a folded piece of muslin dipped in warm water laid over the part. A flannel bandage pinned around the body and kept in position by means of perineal straps completed the dressing. The patient was now removed from the table and placed in bed. Two teaspoonsful of elixir of opium were given her, and as she appeared somewhat prostrated, some brandy and water were also administered.

Ten o'clock P. M., complains of great thirst and some pain in the abdomen. Pulse 100, full, tense. Empty the bladder with the catheter, and give two teaspoonsful of McMunn's elixir of opium. Enjoin strict rest, and allow ice held in the mouth to quench thirst.

September 1st, 9 A. M. Has slept some during the night; pulse 112; tongue slightly furred; complains of thirst, and slight pain in the abdomen; introduce catheter; allow ice and toast-water. 10 P. M. Still has slight pain; empty bladder.

2d, 6 A. M. Expresses herself as feeling very well. Thinks she is able to get out of bed. Pulse 112; empty bladder. Allow ice and toast-water, with a small quantity of cream added to the latter. 12 M. Has been sleeping, feels quite comfortable; pulse 98. Give two teaspoonsful elixir opium. 3 P. M. Has been sleeping; pulse 108; empty bladder. 12 midnight. Comfortable; emptied bladder.

3d, 10 A. M. Slept some during the night; pulse 90; comfortable, and has less thirst. Examine the wound for the first time. It looks well, and the union of the edges appears to be perfect, except at the point where the stumps of the pedicles protrude; the latter appear shrunken and blackened. Apply fresh compresses wrung out of warm water; order weak chicken-water as diet, and ice to allay thirst; empty the bladder. 5 P. M. Pulse 100; has been sleeping, and feels well; tongue slightly coated with a whitish fur, a condition which has been present from the first; has taken the chicken-water several times with enjoyment. 12 midnight. Comfortable. Dress the wound, and give two teaspoonsful elixir opium.

4th, 9 A. M. Pulse 104. Make an entire change of clothing without raising the patient from the bed. The stumps of the pedicles not being removed, apply a flaxseed-meal poultice over that portion of the wound. Order chicken-water with rice, empty bladder, and give full dose of opium. 5 P. M. Comfortable; introduce catheter. 12 midnight. Empty bladder, and give full dose morph. sulph.

5th, 8 A. M. Complains of pain and tenderness in both iliac regions; pulse 100; says the morphia acted unpleasantly, and did not produce sleep. Empty bladder; ordered flannels wrung out of hot water to be kept constantly applied to the abdomen. 1 P. M. Pulse 108; has less pain; appetite good, and takes the broth with great relish. Wound looks well; no swelling of the abdomen. 12 midnight. Comfortable. Empty bladder; give full dose of opium.

6th, 10 A. M. Did not sleep well last night, but feels cheerful this morning. Dress lower portion of the wound, and apply lard to the sutures and pins to their points of ingress and egress; empty bladder; order the quantity of nourisment to be increased. 5 P. M. Has been sleeping soundly, and feels very comfortable; pulse 92; appetite good; no tenderness of the abdomen on slight pressure. Introduce catheter. 12 midnight. Dress lower end of wound; some of the ligatures on the pedicles are loose; empty bladder; omit the opium.

7th, 9 A. M. Pulse 84; has slept well. Dress lower end of wound; introduce catheter; order 1 oz. castor oil, and permit the head and shoulders to be somewhat raised. 4 P. M. The castor oil has produced three copious evacuations without pain. Dress the lower portion of the wound, and remove two of the sutures; introduce catheter. Patient able to move in any direction without pain. Change her clothes and the bedding; afterwards administer a small quantity of brandy. 12 midnight. Not quite so well; the bowels have been moved three more times, occasioning weakness; pulse 88. Empty bladder; dress the wound; give full dose of opium.

8th. 9 A. M. Pulse 100; bowels quite loose, and patient feels weak. Dress lower end of wound, and remove one hare-lip pin; give a cup of strong black tea; introduce catheter. 1 P. M. Bowels have acted four times since last visit. Fearful that such excessive peristaltic action may induce peritonitis; introduce a suppository of morphia into rectum. 4½ P. M. Has been sleeping; pulse 88; bowels quiet since last visit. Empty bladder; order a bowl of strong broth with rice. 12 midnight. Pulse 100; bowels open twice during the evening. Introduce morphia suppository; empty bladder.

9th, 9 A. M. Feels better this morning; pulse 96; bowels not moved since last visit; has had some nausea and vomiting; has taken some coffee and cracker this morning with relish. Dress lower end of wound, and empty bladder. 5 P. M. Pulse 92; bowels moved once. Remove one of the pins that had been passed through the pedicles; introduce catheter. 12 midnight. Pulse 88; feels somewhat uncomfortable from having eaten too freely. Empty bladder; remove two of the wire sutures, and the pin transfixing the other pedicle.

10th, 9 A. M. Feels unusually well; got out of bed this morning and sat on a chair twenty minutes, and emptied the bladder by her own effort; is quite bright and cheerful; pulse 80; a small quantity of fetid pus is discharged when pressure is made on the side of the wound, at the point where the pedicles emerge. 6 P. M. Pulse 80; has been out of bed again to empty the bladder.

11th, 9 A. M. Pulse 76; everything going on favorably. Dress wound carefully, and remove the remainder of the pedicle ligatures. Has been up twice to urinate. 10 P. M. Pulse 72; bowels moved once; appetite excellent; has been up four times during the day.

12th, 9 A. M. Dress the wound. Patient feels quite well this morning. 6 P. M. Still improving; pulse 72; there seems a slight tendency to diarrhœa. Order a morphia suppository.

13th, 9 A. M. Much better. Dress the wound, which is rapidly healing.

From this time the patient continued to improve. At the end of three weeks she left her room and walked down stairs. Her recovery was uninterrupted, and at this time (April 16, 1866) is in as good health as at any former period of her life.

*Remarks.*—I make no apology for detailing the foregoing case so fully, for I apprehend that success in ovariotomy, as in other large surgical operations, frequently depends in very great measure upon attention to the *minutiæ* of the after-treatment. And, wherever it is practicable, I would recommend that the operator himself, in so far as may be possible, superintend the management of the case. The reasons for this advice are sufficiently obvious. Others may do well—friends may furnish such attentions as are always pleasant to the invalid, and skilled attendants may perform every duty—but there is none who feels a more intense anxiety for the recovery of a patient than he who has jeopardized that patient's life in order to preserve it.—*American Journal of the Medical Sciences.*

